# Optical Coherence Tomography Measurements in Type 1 Diabetic Subjects with Low and Moderate Daily Physical Activity

**DOI:** 10.22336/rjo.2023.55

**Published:** 2023

**Authors:** Corina-Iuliana Suciu, Vlad-Ioan Suciu, Simona Delia Nicoară

**Affiliations:** * Department of Ophthalmology, “Iuliu Haţieganu” University of Medicine and Pharmacy, Cluj-Napoca, Romania; ** Department of Neuroscience, “Iuliu Haţieganu” University of Medicine and Pharmacy, Cluj-Napoca, Romania; *** Department of Ophthalmology, Emergency County Hospital, Cluj-Napoca, Romania

**Keywords:** diabetic macular edema, disease prevention, diabetes mellitus complications, physical activity, optical coherence tomography, quality of life

## Abstract

**Background:** Physical activity is nowadays recognized as a protective factor against cardiovascular conditions, being cost-effective and easy to implement. Through its positive effects on hemodynamic and oxidative stress, different intensities in daily physical activity could influence diabetic macular edema (DME) in type 1 Diabetes Mellitus (DM).

**Methods:** With the help of a spectral domain optical coherence tomography (OCT) device, we studied the macular thickness and ETDRS map parameters in type 1 DM patients who were classified into two groups: low and moderate intensity routine physical activity status, using the international physical activity questionnaire (IPAQ). All subjects received comparable anti-VEGF treatment.

**Results:** Having a long disease evolution, patients with type 1 DM (T1DM) with moderate physical activity displayed better OCT measurements in specific retinal sectors than their counterparts with low physical activity. Variables such as age and body mass index (BMI) can influence the level of physical activity in T1DM patients.

**Conclusions:** This study showed a lower prevalence of DME in T1DM subjects with moderate physical activity levels, revealing lower values for ETDRS OCT parameters in specific retinal sectors. The macular volumes (mm3) were significantly lower in the right eye for this group of subjects.

**Abbreviations:** BMI = body mass index, CMT = central macular thickness, DM = diabetes mellitus, DME = diabetic macular edema, DR = diabetic retinopathy, FT = foveal thickness, II = inferior inner thickness, IO = inferior outer thickness, IPAQ = international physical activity questionnaire, LE = left eye, OCT = optical coherence tomography, MMT = maximal macular thickness, mMT = minimal macular thickness, MV = macular volume, NI = nasal inner thickness, NO = nasal outer thickness, QoL = quality of life, RE = right eye, SI = superior inner thickness, SO = superior outer thickness, T1DM = type 1 diabetes mellitus, T2DM = type 2 diabetes mellitus, TI = temporal inner thickness, TO = temporal outer thickness

## Introduction

Diabetic retinopathy (DR) is the most common cause of vision loss in the active population, with Diabetic Macular Edema (DME) representing up to one-third of cases. However, DME is a very common preventable cause of visual loss [**1**-**3**]. Timely diagnosis and prevention through strict glycemic control and correction of other risk factors for progression hold the key to decreasing the prevalence of DME and, at the same time increasing the patients’ quality of life (QoL).

Optical coherence tomography (OCT) is a well-known non-invasive, in-vivo investigation method for many ophthalmological and neurological disorders [**4**,**5**]. Many OCT biomarkers for DME are discovered through studies, which makes the screening methods more accessible, precise, and time-efficient [**6**,**7**]. In a previous study, we identified demographic (male gender, urban environment, non-academic education), clinical (positive family history of DM, obesity, vascular risk factors), and OCT (eccentric pattern of DME, lateralization of DME lesions) risk factors associated with higher severity and progression of DME, stressing the need for a complete evaluation in screening patients with T1DM [**4**].

The International Physical Activity Questionnaire (IPAQ) represents a standardized method of measuring the physical activity in the adult population for a week. According to the intensity of the physical activity, a metabolic equivalent (MET) score is computed by considering the estimated level of energy, duration, and frequency of a specific activity, over one week. Different levels of physical activity result from this equation and correspond to three physical activity intensity categories (low, moderate, and high) [**8**-**10**].

Regular physical activity has well-known benefits for cardiovascular diseases, as well as in controlling blood sugar and lipid metabolism in patients with DM. On the other hand, according to recent studies,the lack of physical activity leads to myocardial infarction, high blood pressure, obesity, and increased insulin resistance [**9**,**11**,**12**]. Simonetti et al. demonstrated that 76.9% of individuals who suffered a myocardial infarction and 81.2% of patients with hypertension had not performed any physical activity [**8**,**11**-**13**]. A prospective 10-year study on 9,018 individuals showed that an increased level of physical activity was independently associated with a lower need for retinal pan-photocoagulation in patients with DM [**14**]. Kleinman et al. showed that intense physical activity lasting 15 minutes increased retinal blood flow [**15**].

The purpose of this research was to investigate the effect of physical activity in patients with T1DM and to check its impact on the macular thickness and ETDRS map OCT parameters.

## Materials and methods

This observational, cross-sectional study evaluated 2 patient groups (n=25 subjects, examining a total of 36 eyes with DME) according to the IPAQ physical activity score (MET1 = low physical activity; MET2 = moderate physical activity): MET1 (n=13), MET2 (n=12), during 3 years (2019-2022). Each patient was assessed according to the following criteria: epidemiologic and demographic factors (age, gender, duration of DM), clinical and anthropometric measurements (ophthalmologic examination, blood pressure, BMI), OCT imaging, laboratory tests (HbA1c, total proteinuria/24 h, BUN, serum creatinine, eGFR, ESR, LDLc, HDLc, Triglycerides) and IPAQ physical activity research tool. The ophthalmologic examination included the following: patient history, best corrected visual acuity (BCVA- Snellen Chart) measurement, anterior and posterior segment examination, and ophthalmoscopic examination on dilated pupils using Tropicamide 1% eye drops. The OCT examinations were performed on a Heidelberg Spectralis® OCT2 device (Heidelberg Engineering, Germany) having the Spectralis Software version 6.10.5 installed for processing. The macular scanning was done with thickness map rendering (20 x 20° with 25 slices per retina at 200 µm; real-time value of 9). Scan and rendering contained the ETDRS macular map, macular volume (MV), maximal macular thickness (MMT), minimal macular thickness (mMT), central macular thickness (CMT), foveal thickness (FT), superior inner thickness (SI), inferior inner thickness (II), nasal inner thickness (NI), temporal inner thickness (TI), superior outer thickness (SO), inferior outer thickness (IO), nasal outer thickness (NO), and temporal outer thickness (TO).

The present study included subjects classified in either MET1 or MET2 scores, according to the IPAQ long-form research tool, which assessed 5 areas of daily activities such as job-related, transportation, housework, leisure-time physical activities, and the time spent sitting. MET1 was defined by a lack of minimal physical activity, with a total MET score of <600 METs/min/week. MET2 was considered a moderate intensity of physical activity with >30 minutes of intense physical activity/day for at least 3 days or moderate physical activity/day for 5 days or any combination of physical activity for 5 days and 600-1500 METs/min/week. For example, a moderate physical activity quantifies 4 METs and an intense physical activity quantifies 8 METs. In our study, we considered the total MET score per week for everyone.

All subjects were aged over 18 years and Caucasians. Recruitment of subjects in the study was based upon voluntary participation. Age stratification was performedto obtain comparable results and control confounders. The exclusion criteria were: the association of age-related macular degeneration, open-angle glaucoma, retinal vein occlusions, or optic neuropathies. All subjects were diagnosed with DME in one or both eyes and all were treated with intravitreal anti-VEGF agents (Bevacizumab) per the guidelines. DME was defined by OCT as having CMT ≥270 µm, an MV ≥8.59 µm3, and at least one OCT morphologic marker (intraretinal/ subretinal fluid, ellipsoid zone (EZ) disruption, hyperreflective foci or disorganization of the inner retinal layers).

To control confounders and reduce bias, both eyes were individually investigated throughout the 2 study groups to fully evaluate both retinas.

Concerning ethics and good clinical practices, all enrolled subjects signed an informed consent. All subjects were recruited from the Department of Ophthalmology, Emergency County Hospital, Cluj-Napoca, Romania. The study was approved by the Ethics Committee of the “Iuliu Haţieganu” University of Medicine and Pharmacy, Cluj-Napoca, Romania (347/1.10.2019) and the Emergency County Hospital, Cluj-Napoca, Romania (37067/19.12.2019). The study adheres to the Declaration of Helsinki.

Microsoft Office Professional Plus 2016 (64-bit version, Microsoft, Redmond, WA, USA) and SPSS Statistics V.28.0.1 (SPSS Inc., Chicago, IL, USA) software were used for statistical analyses. ANOVA, t-test, Kolmogorov-Smirnov test, Chi-Square test, multiple regression analysis, and F-Test were conducted. A p-value of <0.05 was set for statistical significance.

## Results

*Physical activity and clinical measurements*

The mean age of the subjects was 58±11 years with male predominance (M/F = 1.5/1). **Table 1** summarizes the physical activity, clinical and laboratory findings.

**Table 1:** Physical activity and clinical measurements

	MET1 (Mean±SD)	MET2 (Mean±SD)	*P* Value MET1 vs. MET2
Disease duration (years)	17.4±6.2	26.6±11.8	0.01*
MET IPAQ score	483.4±92.2	1053.3±312.6	<0.0001*
Anti-VEGF injections (n)	4±1.4	3.3±2.4	0.49*
BMI kg/m2	32.2±4.8	26.1±4.3	0.001*
Overweight	38%	25%	0.24*
Obese	62%	25%	<0.01*
Normal weight	0%	50%	<0.0001*
HbA1c g%	9.1±1.7	9.8±2	0.2*
Total proteinuria/ 24h	39.5±45.5	42.1±46.6	0.44*
BUN mg%	73.3±45	52±30.3	0.1*
Serum creatinine mg%	1.6±1.2	2±2.1	0.26*
eGFR mL/min/ 1.73 m2	57.9±29.4	64.1±32.8	0.31*
ESR mm/h	29.6±16	15.9±18	0.03*
Dyslipidemia	69% present	75% present	0.74***
LDLc mg%	99.4±21.1	110.5±36.2	0.18*
HDLc mg%	41.8±11.8	46.2±17.5	0.23*
Triglycerides mg%	178.3±80.5	183.3±172.2	0.46*
SBP mmHg	147.5±16.5	143±26.2	0.3*
DBP mmHg	83.9±9.3	82.5±11.9	0.37*
MBP mmHg	105.1±10.2	102.6±14.8	0.31*
Urban environment	69%	67%	0.89***
significance p <0.05; *ttest; **ANOVA; ***Chi-Square Test			

The disease duration was significantly different between the MET groups, being longer in the MET2 group (26.6±11.8 years; p = 0.01). The mean number of intravitreal anti-VEGF injections with bevacizumab was similar between the groups (p = 0.49).

Significantly different mean IPAQ-MET scores were found between both comparison groups (p = 0.0001). The body mass index (BMI) was significantly lower in the MET2 group, with more obese individuals being found in the MET1 group and most normal-weightindividuals, in the MET2 group.

**Fig. 1:**
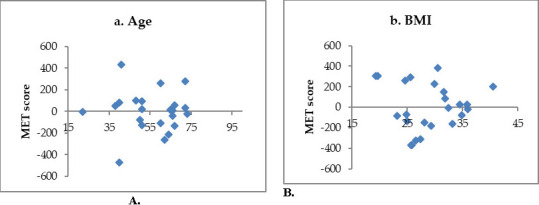
Residual plots for the MET score and independent variables in T1DM (**A,B**)

A multivariable linear regression analysis was done to evaluate the effect of age, gender, disease duration, HbA1c, and BMI on the physical activity MET score. In this model, a relationship was shown between the MET score and age (p <0.0001; Coefficient = -16.9; 95% CI -23.9, -9.9) but also between the MET score and BMI (p =0.001; Coefficient = -26.7; 95% CI -42.2, -11) (**Table 2**). The scatter-residual plots for T1DM subjects (**Fig. 1 A,B**) illustrate the relative homogeneity of variance of the two variables (age and BMI) about the MET score.

**Table 2:** The relationship between the physical activity (MET score, reference variable) and multiple independent variables (predictors)

Predictors	Regression Coefficient (95% CI)	*p*-Value
BMI (kg/m2)	-26.7 (-42.2, -11)	0.001
HbA1c (g%)	-8.3 (-45, 28.3)	0.63
Disease duration (years)	2.4 (-3.9, 8.9)	0.43
Gender (male)	-58.5 (-197.5, 80.4)	0.38
Age (years)	-16.9 (-23.9, -9.9)	<0.0001
	Adjusted R2 = 83%	
*BMI = body mass index; CI = Confidence Interval; statistical significance threshold p <0.05*		

*The ocular and OCT parameters concerning physical activity*

In terms of the OCT parameters, MET2 had a significantly lower prevalence of DME in both eyes (p = 0.02), when compared to MET1.

The MV (mm3) was significantly lower in the MET2 group (p = 0.04) only for the right eye (RE), whereas regarding the left eye (LE) no significant differencewas observed. The LE showed less ellipsoid zone disruption in the MET2 group (25%; p = 0.001) when compared to the MET1 group (77%).

Concerning the retinal sectors, the MET2 group showed significantly lower values of the MMT (p = 0.01), mMT (p = 0.008), CMT (p = 0.05), FT (p = 0.01), SI thickness (p = 0.01), NI thickness (p = 0.01), TI thickness (p = 0.04) and TO thickness (p = 0.04), when compared to the MET1 group for the RE. The LE showed a significant difference only for the central thickness between MET1 and MET2 (p = 0.04). Regarding the BCVA, a significant statistical difference was observedbetween groups in both eyes, with better vision within the MET2 group. **Table 3** summarizes the mean OCT values and compares each group pair.

**Table 3:** OCT Parameters comparison between groups

			MET1 (Mean±SD)	MET2 (Mean±SD)	*P* Value MET1 vs. MET2
BCVA		RE	0.32±0.28	0.6±0.27	0.02*
		LE	0.16±0.14	0.47±0.34	0.006*
DME in both eyes			69%	42%	0.02***
EZ lesion		RE	54%	50%	0.41***
		LE	77%	25%	0.001***
MV (mm3)		RE	11.6±2.8	9.6±1.1	0.04*
		LE	10.2±1.4	9.7±1.5	0.25*
RE ETDRS map OCT parameters		MMT	568.5±209.2	391.8±79.4	0.01*
		mMT	403.8±156.2	258.8±77.3	0.008*
		CMT	463.3±197.7	311.5±138.6	0.05*
		FT	481.2±177.2	316±73.7	0.01*
	Superior	Inner	509.4±160.3	366.6±33.7	0.01*
		Outer	429.4±140.6	331.5±56.4	0.04*
	Inferior	Inner	439.6±125.1	376.2±22.7	0.07*
		Outer	353.5±72.2	327.8±40.8	0.17*
	Nasal	Inner	466±113	374.1±20.4	0.01*
		Outer	389±72.6	349.3±32.7	0.07*
	Temporal	Inner	482±155.7	386.2±55.3	0.04*
		Outer	409.6±114.8	348.6±54.9	0.08*
LE ETDRS map OCT parameters		MMT	489.9±160.4	381.8±102.6	0.05*
		mMT	326.4±135.4	260.7±79.6	0.11*
		CMT	377.5±170.7	268.1±78.9	0.04*
		FT	407.6±149.1	313.7±85	0.06*
	Superior	Inner	405.6±102.8	379.5±71.7	0.28*
		Outer	347.8±70.4	328.5±50.7	0.27*
	Inferior	Inner	412.9±100.2	378.5±89.1	0.23*
		Outer	350.3±77.5	325.5±63.7	0.24*
	Nasal	Inner	411.2±114.7	374.7±74	0.23*
		Outer	358.6±71.9	347.5±57.8	0.36*
	Temporal	Inner	412.5±116.8	368.4±55.4	0.18*
		Outer	356.3±83.1	325.7±40.8	0.19*
BCVA = best corrected visual acuity; SD = standard deviation; RE = right eye; LE = left eye; MV = macular volume; EZ = Ellipsoid zone; ETDRS = Early Treatment Diabetic Retinopathy Study; MMT = Max. macular thickness; mMT= Min. macular thickness; CMT = Central macular thickness; FT = Foveal thickness; SI = Superior inner thickness; II = Inferior inner; NI = Nasal inner; TI = Temporal inner. Significance p <0.05; *ttest; **ANOVA; ***Chi-Square test.					

## Discussions

T1DM represents 5-10% of all types of DM, having a peak incidence of diagnosis around 10 to 14 years of age [**16**-**18**]. Therefore, these patients may have a longer disease duration compared to T2DM patients. Despite this, the pharmacological anti-VEGF treatment was comparable, showing no significant statistical differences between both groups, determining a better comparison of the effect of physical activity on DME.

The regression analysis revealed a statistically significant association between age and BMI for the physical activity status in T1DM subjects. This model predicts that for each year of aging in T1DM subjects, the MET score decreases by -16.9 points. Also, for each BMI unit (kg/m2) increase, the MET score decreases by -26.7 points. This translates into the fact that there is a tendency for decreased physical activity in older and overweight/obeseT1DM subjects. Based on these observations, age and BMI can influence the physical activity score and possibly, indirectly the DME outcome in T1DM patients. The interdependence of physical activity and BMI is noteworthy, as these variables can influence each other. Yan X et al. confirm our findings, showing that the association between physical activity and DR progression was influenced by factors such as gender and BMI [**14**]. Further research is needed to confirm our observations.

In the present study, we showed that male gender was predominant in the MET2 group. In a study on a mixed geographical population including 510 subjects, 36.8% were classified in the highly active IPAQ physical activity score, while 17.8% were inactive. The authors did not find any significant difference between IPAQ scores in the male population with and without DM [**19**]. Another study published by Yan et al. showed that DR progression was slower in the male population with significant physical activity, possibly due to either longer periods of physical activity or higher intensity. The benefits of the physical activity were demonstrated only in T2DM, not in T1DM [**14**]. Wang et al. documented the effects of physical activity on various ocular diseases (cataracts, glaucoma, retinal vein occlusion, central serous choroidopathy, age-related macular degeneration, and DR) in a Chinese population and found that the prevalence of DR was significantly lower in subjects with high physical activity levels. Moreover, the only ocular condition thatwas influenced by the physical activity status was DR [**20**]. Through clinical and mostly OCT parameters our study revealed that moderate physical activity was associated with lower macular thicknesses in T1DM in a male-predominant sample.

In the MET2 group, a significantly longer disease duration was shown, when compared to the MET1 group. Despite this, better eGFR values were foundin the MET2 compared to the MET1 group, although not statistically significant. A meta-analysis showed the importance of physical activity in decreasing the incidence of diabetic nephropathy in T1DM subjects. Moreover, evidence showed that physical activity has positive effects on the lipid profile and endothelial function, which in turn reduces glomerular and tubulointerstitial injuries [**21**].

Other authors confirmed the decrease of HbA1c in T2DM by sustained exercise for over 150 minutes per week [**22**,**23**]. Physical activity was demonstrated to lower the incidence of T2DM, especially in subjects with modified glucose tolerance, being even more effective than treatment with metformin. Many studies on animal models and humans revealed that chronic inflammation and secondary endothelial dysfunction in DM are related to the development and progression of DR. Ong et al. concluded that physical activity could protect against visual dysfunction.The possible mechanism could be represented by the change in the caliber of the central retinal vein during physical exercise. Moreover, they found that high physical activity could determine the narrowing of the retinal venous caliber and arterio-venous nicking [**24**]. Our study could not demonstrate a significant difference between the MET groups in T1DM subjects, possibly because physical activity reduces insulin resistance and not insulin secretion as in T1DM.

Best mean BMI values with the fewest obese patients and the most normal weight patientswere observed in the MET2 group, demonstrating the beneficial effects of physical activity on weight control, as shown by other authors [**8**,**9**,**21**]. Obese patients with low physical activity were linked to displaying microvascular diabetic changes in one study on 977 subjects from Bangladesh (p <0.001) [**25**].

The inflammation marker (ESR) was lower in the MET2 group, with statistical significance (p = 0.03). It has been shown that regular physical activity can modulate oxidative stress and inflammation [**6**,**26**-**32**]. Animal experiments revealed that changing the pro-inflammatory microglia to an anti-inflammatory phenotype, through treadmill exercise, resulted in reducing the severity of DR [**26**,**27**].

The MET 2 group presented a lower prevalence of DME in both eyes, with statistically significant results (p = 0.02). The MET2 group displayed the lowest mean macular volume (mm3) for the RE despite having the longest disease duration. Moreover, fewer ellipsoid zone disruptions were observed via OCT imaging in the MET2 group.

The physical activity showed a beneficial effect on the evolution of DME within the T1DM groups, with lower values (µm) in the RE for the MMT, mMT, CMT, FT, SI thickness, NI thickness, TI thickness, and superior outer sectors. These values were also lower in the LE but with statistically significant results only for the CMT.

Concerning the BCVA measurements, the mean BCVA values showed better vision within the MET2 group of both eyes with statistically significant differences. This observation is consistent with the OCT parameters such as central macular thickness and EZ disruption in both eyes. Although OCT reveals microstructural changes in the retina and BCVA measures the visual function, it has been demonstrated in previous studies that BCVA is not a reliable marker for measuring the outcome in DME patients. Instead, the OCT parameters have been shown to effectively quantify the severity and evolution of DME with or without treatment [**7**,**33**,**34**]. Therefore, we focused our research on the OCT parameters in evaluating the morphology of the retina in DME subjects with two types of physical activity intensities daily, even though the BCVA confirmed the OCT differences between groups. Sharma et al. [**33**] studied the treatment response to Bevacizumab in different DME patterns and measured the outcomes through OCT parameters (central macular thickness) and BCVA. The response measured via OCT had a statistically significant difference among groups, while no significant variation could be demonstrated by the BCVA parameters alone, showing that BCVA was not a reliable marker for measuring the effect of Bevacizumab treatment in DME. Bing Li et al. [**34**] studied the quantitative correlation between the OCT parameters and BCVA, demonstrating that BCVA is not a surrogate marker for DME, as BCVA can be influenced by miscellaneous factors. Moreover, the OCT parameters could explain only 52-60% of the BCVA variation.

The mechanisms involved in the prevention and regression of DR and related microvascular complications by physical activity are not fully understood. Beneficial effects could be represented by the hemodynamic changes and the reduction of oxidative stress causing the retrogression of oxidative processes. In a recent study,Yan et al. demonstrated a negative correlation between the severity of DR and physical activity based on a 10-year study. However, no reference is madeto the influence of physical activity on the evolution of DME [**14**]. Alten et al. studied the effect of a high-intensity training exercise on choriocapillaris circulation in young patients with T1DM without DR and compared the results to healthy controls. Angiographic images revealed a flow deficit in subjects with T1DM (p <0.001) [**35**].

A study on a diabetic animal model using Wistar rats showed that exercise training improves the control of baro- and chemo-reflexes in arteries. These observations strengthen the importance of physical activity in reducing cardiovascular risk and maintaining optimal circulation in individuals with DM [**35**,**36**]. Freitas et al. confirm these findings based on a study that was carriedout on rats with DM, trained for 10 weeks on a treadmill. The blood flow was measured using microspheres. They demonstrated the improvement of blood flow, vascular resistance, and reduction of the long-term organ dysfunctions induced by hyperglycemia inexercise-trained rats [**37**]. Yet another study revealed the beneficial effects of physical exercise on diabetic rats that were active before the onset of DM, with an impact on reducing oxidative stress and improving renal function [**38**]. A large, 8-year study performed on T2DM subjects in Taiwan showed that renal protection and albuminuria remission were protective factors against the development of DME and proliferative DR [**39**]. According to the above-mentioned studies, the increase of blood flow during physical activity leads to the improvement of renal function, decrease of albuminuria, and regression of DME.

AlQabandi and colleagues recognize the importance and beneficial effect of physical activity on public health concerning high blood pressure, DM, and neoplasia. They consider that physical activity is not only a cost-effective therapy for reducing the incidence of the above-mentioned conditions but also within reach for everyone. They also state that the lack of physical activity is a risk factor for DM and related complications (chronic renal disease, retinopathy). Most patients with T1DM develop DR within the first 20 years of disease, while 60% of patients with T2DM already have DR at the time of DM diagnosis [**40**].

## Conclusions

Nowadays, the importance of physical activity is increasingly more recognized in the community as having a beneficial role in disease prevention and in improving patient outcomes. To the best of our knowledge, this is the first study that focuses on evaluating the effect of physical activity, through a standardized scoring system (IPAQ - MET), on the OCT parameters in DME for patients with T1DM.

This study showed a lower prevalence of DME for both eyes in T1DM subjects with moderate physical activity, revealing lower values for OCT parameters in specific retinal sectors (RE MMT, mMT, FT, SI, NI, TI, Superior outer, and LE CMT). Moreover, the macular volumes (mm3) were significantly lower in the RE for the MET2 group. This could signify the importance of moderate physical activity in improving specific OCT measurements for patients with T1DM.

The present study was limited by a relatively small sample size and evaluation period (3 years), being done in only one research center, and by the fact that two variables such as age and BMI were shown to have a relationship with the physical activity status in T1DM subjects. Nevertheless, other variables could not demonstrate any impact on physical activity. Even so, the data should be interpreted in the present context. Because of the paramount importance of physical activity for public health and healthcare costs, further research is needed to confirm our findings and improve patient QoL.

The practical outcome of our research is the stressing of the importance of physical activity as a cost-effective and easy-to-implement complementary intervention associated with the pharmacological treatment (anti-VEGF) for DME tomaximize the QoL for DME patients, especially in the working population, as DME greatly affects the macular OCT parameters in this category. Therefore, the medical staff and healthcare providers are encouraged to consider this complementary therapeutic option in all subjects with DM.

**Conflict of Interest Statement**

The authors state no conflict of interest. 

**Informed Consent and Human and Animal Rights Statement**

Informed consent has been obtained from all the patients included in the study.

**Authorization for the use of human subjects**

Ethical approval: The research related to human use complies with all the relevant national regulations and institutional policies, it is per the tenets of the Helsinki Declaration and has been approved by the review board of “Iuliu Haţieganu” University of Medicine and Pharmacy, Cluj-Napoca, Romania. 

**Acknowledgments**

All authors contributed substantially to this manuscript. We thank all our patients who participated in this study and devoted their time to knowledge.

**Sources of Funding**

No sources of funding to declare.

**Disclosures**

None.
